# Pan‐cancer analysis reveals the oncogenic role of 3‐hydroxy‐3‐methylglutaryl‐CoA synthase 1

**DOI:** 10.1002/cnr2.1562

**Published:** 2021-09-22

**Authors:** Cheng Zhou, Zhiqin Wang, Yueqing Cao, Liang Zhao

**Affiliations:** ^1^ Department of Hematology Xiangya Hospital, Central South University Changsha Hunan China; ^2^ Department of Geriatric Neurology Xiangya Hospital, Central South University Changsha Hunan China

**Keywords:** cancer, drug sensitivity, genetic alteration, HMGCS1, mevalonate pathway, prognosis

## Abstract

**Background:**

Emerging studies reveals that 3‐hydroxy‐3‐methylglutaryl‐CoA synthase 1 (HMGCS1) plays vital oncogenic roles in a broad spectrum of human cancers, but there is no pan‐cancer evidence on the relationship between HMGCS1 and various tumor types.

**Aim:**

To explore the potential role of HMGCS1 across various tumor types based on big clinical data.

**Methods:**

We conducted a pan‐cancer analysis across more than 30 tumor types, based on the most comprehensive database available, including TCGA, GSCA, clinical proteomic tumor analysis consortium, Kaplan–Meier Plotter dataset, GEPIA2, TIMER2, STRING, and GDSC dataset.

**Results:**

HMGCS1 was highly expressed and negatively correlated with the prognosis in most cancer types. The infiltration levels of cancer associated fibroblast and CD8+ T‐cell were closely associated with HMGCS1 expression. Amplification was the most common genetic alteration of HMGCS1 in different cancers, while the frequency of mutation was low. Besides, ACAT2 and MVD were closely correlated and bind to HMGCS1. Pathway enrichment analysis indicated that HMGCS1 was actively involved in steroid biosynthesis. Moreover, high HMGCS1 expression could reduce the sensitivity to most drugs in the GDSC dataset.

**Conclusions:**

Our study revealed the potential oncogenic role of HMGCS1 in cancers.

## 1 INTRODUCTION

The etiology of cancer is complicated, with both genetic and epigenetic alterations involved. It is significant to conduct a pan‐cancer expression analysis of particular gene of interest and assess its correlation with diagnosis, treatment, prognosis, and potential molecular mechanisms.^1^, ^2^

The mevalonate (MVA) pathway is known as synthesizing sterols and isoprenoids, playing a key role in multiple cellular processes.^3^, ^4^ Recent studies highlighted the importance of MVA metabolism in cancer, especially the intermediates involved in this pathway.^3^, ^5^, ^6^, ^7^ For instance, cholesterol, an essential component of most cellular membranes, provides building blocks for highly proliferative cancer cells. Cholesterol also contributes to drug resistance and affect immune surveillance in cancer.^8^, ^9^ Besides, farnesyl‐diphosphate and geranylgeranyl diphosphate are requisites for protein isoprenylation, which are vital for intracellular signaling. For example, after isoprenylation, small GTPases such as RAS are transferred to cell membrane and exert oncogenic function of transforming cells.^10^

The 3‐hydroxy‐3‐methylglutaryl‐CoA reductase (HMGCR) is the rate‐limiting enzyme in the MVA pathway. However, other enzymes in the pathway have also been showed to work as controlling points.^7^, ^11^ The 3‐hydroxy‐3‐methylglutaryl‐CoA synthase 1 (HMGCS1) catalyzes the synthesis of HMG‐CoA, which is the substrate for HMGCR.^12^, ^13^ Our previous study has proved that HMGCS1 promoted cell growth in leukemia (data not shown) and BRAF^V600E^‐positive human cancers.^14^ Emerging studies showed that HMGCS1 contributed to the progression of a variety of cancers, including gastric, colon, and cervical cancer.^15^, ^16^, ^17^ Further, the high HMGCS1 expression level was correlated with poor prognosis in breast cancer.^18^ However, there is no pan‐cancer evidence on the relationship between HMGCS1 and various tumor types based on big clinical data.

In this study, we conducted a pan‐cancer analysis across more than 30 tumor types, based on the most comprehensive database available. Our study revealed the potential oncogenic role of HMGCS1 in cancers.

## 2 MATERIALS AND METHODS

### 2.1 Gene expression analysis

By using the “search‐expression” module of ATACdb, a comprehensive human chromatin accessibility database (http://www.licpathway.net/ATACdb/), the expressions of HMGCS1 in primary/nonprimary cells and normal tissues were displayed from NCBI GEO/SRA database. In the “Expression” module of GSCA (Gene Set Cancer Analysis, http://bioinfo.life.hust.edu.cn/GSCA/), we analyzed the HMGCS1 expression data from The Cancer Genome Atlas (TCGA) program, which was obtained from both tumor and adjacent normal tissues of various tumors or tumor subtypes. For some tumors without or with only a few normal tissues, we analyzed the difference of HMGCS1 expression between the tumor and the corresponding normal tissues by GEPIA2 (Gene Expression Profiling Interactive Analysis, version 2, http://gepia2.cancer-pku.cn/). The “expression analysis‐box plots” module was used. In addition, we presented the HMGCS1 expression in every pathological stage (stage I–stage IV) of TCGA tumors by violin plots using the “pathological stage plot” module of GEPIA2.

To compare the level of HMGCS1 protein in tumors and normal tissues, we utilized the UALCAN portal (http://ualcan.path.uab.edu/analysis-prot.html) to analyze the data from the clinical proteomic tumor analysis consortium (CPTAC) dataset.

### 2.2 Survival prognosis analysis

The correlation between HMGCS1 expression and prognosis of tumors was presented by Kaplan–Meier plotter (http://www.kmplot.com/analysis/) and the GEPIA2 database. We calculated the log‐rank *p* value and hazard ratio (HR) with 95% confidence intervals.

### 2.3 Immune infiltration analysis

We utilized the “immune‐gene” of TIMER2 (http://timer.cistrome.org/) to evaluate the correlation of HMGCS1 expression with immune infiltration (IF) across all tumors in TCGA project. The “Gene_Corr” module of TIMER2 was employed to investigate the association between *HMGCS1* expression and IF in different cancer types and in adjacent normal tissues of the TCGA project. Correlations between *HMGCS1* expression and IF were analyzed using the purity‐adjusted partial Spearman's correlation test. The data was displayed by heatmaps and scatter plots. CIBERSORT, CIBERSORT‐ABS, EPIC, MCPCOUNTER, QUANTISEQ, TIDE, and XCELL algorithms were used. The infiltrated immune cells include cancer‐associated fibroblasts (CAF) and CD8+ T cells.

### 2.4 Genetic alteration analysis

We utilized the cBioPortal data (https://www.cbioportal.org/) to look at HMGCS1 alterations and clinical outcomes in the TCGA pipeline. We obtained the alteration frequency, mutation type, and copy number alteration of the HMGCS1 gene via the “cancer types summary” module of cBioPortal. The mutated site information of HMGCS1 was displayed either in the schematic diagram of the protein structure or in the 3D structure using the “mutations” module. The “comparison” module was used to acquire the mRNA and protein expression differences between the TCGA cancer cases with or without HMGCS1 genetic alteration. The heatmaps of HMGCS1 mRNA expression were generated by the TBtools. The Venn diagram was generated by the Xiantao web (https://www.xiantao.love). The “Comparison” module of cBioPortal was employed to explore the correlation between *HMGCS1* genetic alterations and prognoses, including overall survival (OS), progress free survival (PFS), and disease‐free survival (DFS). We built the bivariate linear regression models to study the relationship between HMGCS1 mutations and expression levels.

### 2.5 HMGCS1‐related gene enrichment analysis

We used the STRING (https://string-db.org/) database to analyze the protein–protein interactions (PPI) of HMGCS1. Using the STRING website, we obtained a list of top‐50 HMGCS1‐binding proteins by setting the following parameters: minimum required interaction score (“low confidence [0.150]”), meaning of network edges (“evidence”), max number of interactors to show (“no more than 50 interactors” in first shell), and active interaction sources (“experiments”). GEPIA2 was utilized to obtain the top 50 HMGCS1‐correlated targets. A pairwise gene Pearson correlation analysis was performed. A heatmap was generated by the TIMER2. To analyze the intersection between the HMGCS1‐binding and interacted genes, the Venn diagram viewer of the Xiantao web (https://www.xiantao.love) was utilized. Besides, Kyoto encyclopedia of genes and genomes (KEGG) and Gene Ontology (GO) enrichment analysis were analyzed using the “KEGG/GO enrichment visualization” tool of XianTao.

### 2.6 Drug sensitivity analysis

We performed an unbiased comparison of the drug screening data using GSCA, which integrated over 10 000 genomic data in 33 cancer types from TCGA and over 750 small molecule drugs from genomics of drug sensitivity in cancer (GDSC) and cancer therapeutics response portal.

## 3 RESULTS

### 3.1 . The expression of HMGCS1 in cancers

**FIGURE 1:**
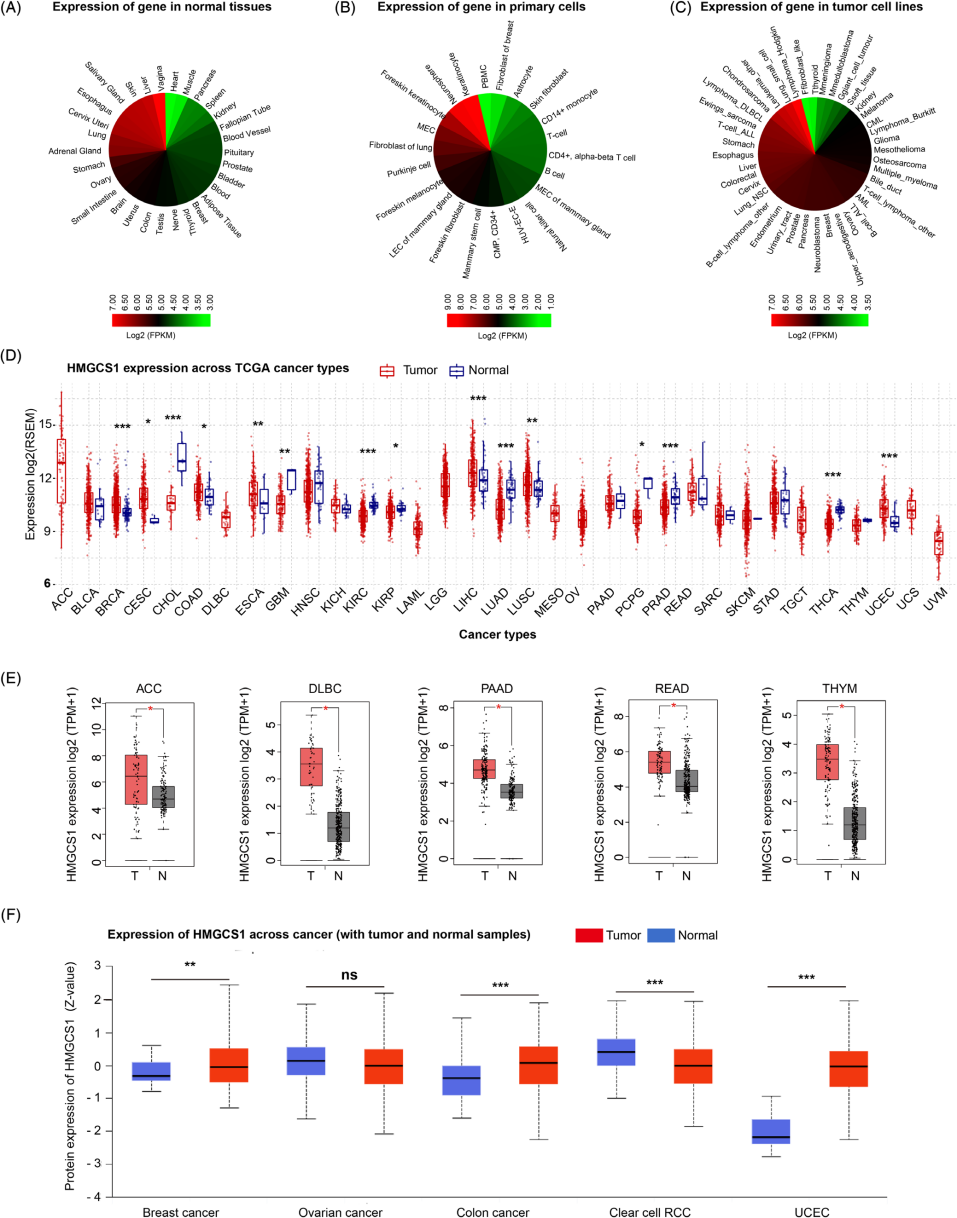
The expression of HMGCS1 in cancers and normal tissues. (A–C) Heatmap of HMGCS1 expressions in normal tissues (A), primary cells (B), and nonprimary cell lines (C). (D) Differential HMGCS1 mRNA expressions between normal tissues and various cancers in the GSCA database. *p < .05; **p < .01; ***p < .001. (E) The levels of HMGCS1 mRNA in tumor tissues and corresponding normal tissues. *p < .01. (F) HMGCS1 total protein levels of normal and primary tissues of breast cancer, ovarian cancer, colon cancer, clear cell RCC and UCEC

First, we studied the expression of *HMGCS1* in various primary/nonprimary cells and normal tissues based on ATACdb database. HMGCS1 was expressed abundantly in normal tissues. The top five normal tissues with highest HMGCS1 mRNA expression were vagina, liver, skin, salivary gland, and esophagus (Figure 1A). In primary cells, keratinocyte cells had the highest HMGCS1 expression, followed by neurosphere, foreskin keratinocyte, mucoepidermal cells (MEC) and fibroblast of lung cells. However, the expression was low in various blood cells, including PBMC, CD14+ monocyte, T‐cell, CD4+ alpha‐beta T cell, natural killer cell, B cell, and common myeloid progenitor CD34+ cell (Figure 1B). Interestingly, we found that HMGCS1 was obviously over‐expressed in tumor cell lines, especially in hematological malignancies and sarcoma (Figure 1C).

Next, we analyzed the HMGCS1 expression of normal and tumor tissues from GSCA approach across different cancer types in TCGA (Figure 1D). Compared to adjacent control tissues, HMGCS1 was over‐expressed in the tumor tissues of breast invasive carcinoma (BRCA), cervical squamous cell carcinoma (CSCA) and endocervical adenocarcinoma (CESC), colon adenocarcinoma (COAD), esophageal carcinoma (ESCA), liver hepatocellular carcinoma (LIHC), lung squamous cell carcinoma and uterine corpus endometrial carcinoma (UCEC), and lower in tumor tissues of cholangiocarcinoma (CHOL), glioblastoma multiforme, kidney renal clear cell carcinoma (KIRC), kidney renal papillary cell carcinoma (KIRP), lung adenocarcinoma (LUAD), pheochromocytoma and paraganglioma (PCPG), prostate adenocarcinoma, and thyroid carcinoma (THCA) (Figure 1D). Furthermore, we used the data of normal tissue from the GTEx dataset as controls to compare the HMGCS1 expression between the tumor and normal tissues. We found that HMGCS1 level was significant higher in adrenocortical carcinoma (ACC), diffuse large B‐cell lymphoma, pancreatic adenocarcinoma, rectum adenocarcinoma (READ), and thymoma (THYM) tumor tissues (Figure 1E), and lower in skin cutaneous melanoma and acute myeloid leukemia (AML) tumor tissues, compared with normal tissues (Figure S1A). However, for other tumors such as LHIC (LGG), ovarian serous cystadenocarcinoma (OV), testicular germ cell tumors (TGCT), and uterine carcinosarcoma (UCS), the expression of HMGCS1 was not significantly different from their controls (Figure S1B). Using the pathological stage plot module of GEPIA2, we observed significantly negative correlation between HMGCS1 expression and pathological stages of KIRC, OV, and THCA (Figure S1C).

In addition, CPTAC dataset was utilized to analyze the HMGCS1 protein level difference among cancers. Compared to normal tissues, HMGCS1 protein was higher in the primary tissues of breast cancer, colon cancer, and UCEC, but was lower in clear cell RCC (also known as KIRC) (Figure 1F).

### 3.2 The correlation of HMGCS1 expression with prognosis in cancers

**FIGURE 2:**
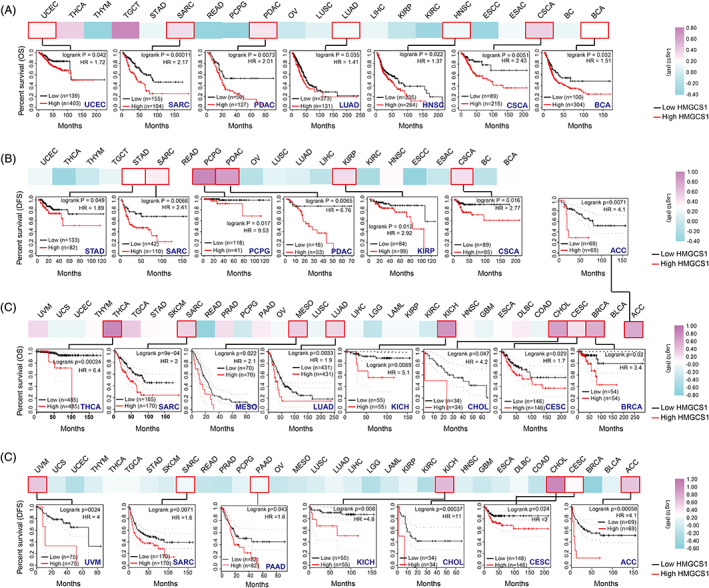
The correlation between the HMGCS1 expression and the prognosis in cancers. (A, B) The correlation between HMGCS1 expression level and overall survival (OS) and disease‐free survival (DFS) in different tumors. (C‐D) The correlation between HMGCS1 expression level and OS (C) and DFS (D) in the GEPIA2 databases. HR, hazard ratio

We utilized the Kaplan–Meier lotter dataset and GEPIA2 to investigate the correlation between HMGCS1 level and OS/DFS. In Kaplan–Meier Plotter dataset, we found that higher level of HMGCS1 was linked to shorter OS in multiple tumor types, including BCA (bladder carcinoma), CSCA, HNSC, PDAC, SARC, UCEC, BRCA, CESC, CHOL, KICH, LUAD, MESO, and THCA (Figure 2A, C). However, highly expressed HMGCS1 was correlated with better prognosis in a few types of tumors including LGG, READ, and KIRC (Figure S2A). Similarly, HMGCS1 expression was negatively correlated with the DFS of ACC, CESC, KIRP, PDAC, PCPG, SARC, STAD, CHOL, KICH, and UVM (Figure 2B, D), but positively correlated with the DFS of KIRC and LGG (Figure S2B). Collectively, HMGCS1 expression was negatively correlated with both OS and DFS in CSCA, PDAC, SARC, ACC, CESC, CHOL, and KICH, while positive associations were found in LGG and KIRC. In conclusion, high expression of HMGCS1 was correlated with poor prognosis in most tumor types, but good prognosis for a small number of tumors.

### 3.3 The correlation between HMGCS1 expression and IF in cancers

**FIGURE 3:**
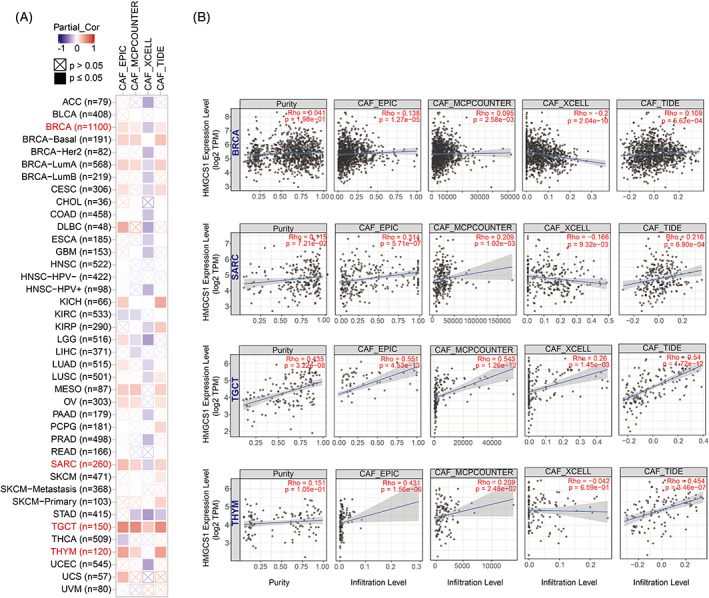
The correlation between HMGCS1 expression and the immune infiltration (IF) in cancers. (A) Using four algorithms, we estimated the correlation between HMGCS1 expressions and IF level of four cancer associated fibroblast cell types across TCGA cancer types. Statistically significant positive/negative correlation is represented by the solid red/blue square, respectively. (B) Some examples of scatter plots show the correlation between HMGCS1 expression and tumor purity (first column), and the infiltration level of cancer associated fibroblast cell estimated by EPIC (second column), MCPCOUNTER (third column), XCELL (fourth column) and TIDE (fifth column), in BRCA (first row), SARC (second row), TGCT (third row), and THYM (fourth row), respectively

HMGCS1 expression was negatively correlated with the IF of CD8+ T‐cells in most cancers, such as CESE, HNSC, STAD, and THYM (Figure S3A, B). In addition, HMGCS1 expression was positively correlated with the estimated infiltration value (EIV) of CAF in the TCGA tumors of BRCA, BRCA−basal, BRCA−LumA, SARC, TGCT, THYM, MESO, and OV (Figures 3A, B). Collectively, we found that the expression of HMGCS1 was correlated with the IF in most cancers.

### 3.4 Genetic alterations of HMGCS1 in cancers

**FIGURE 4:**
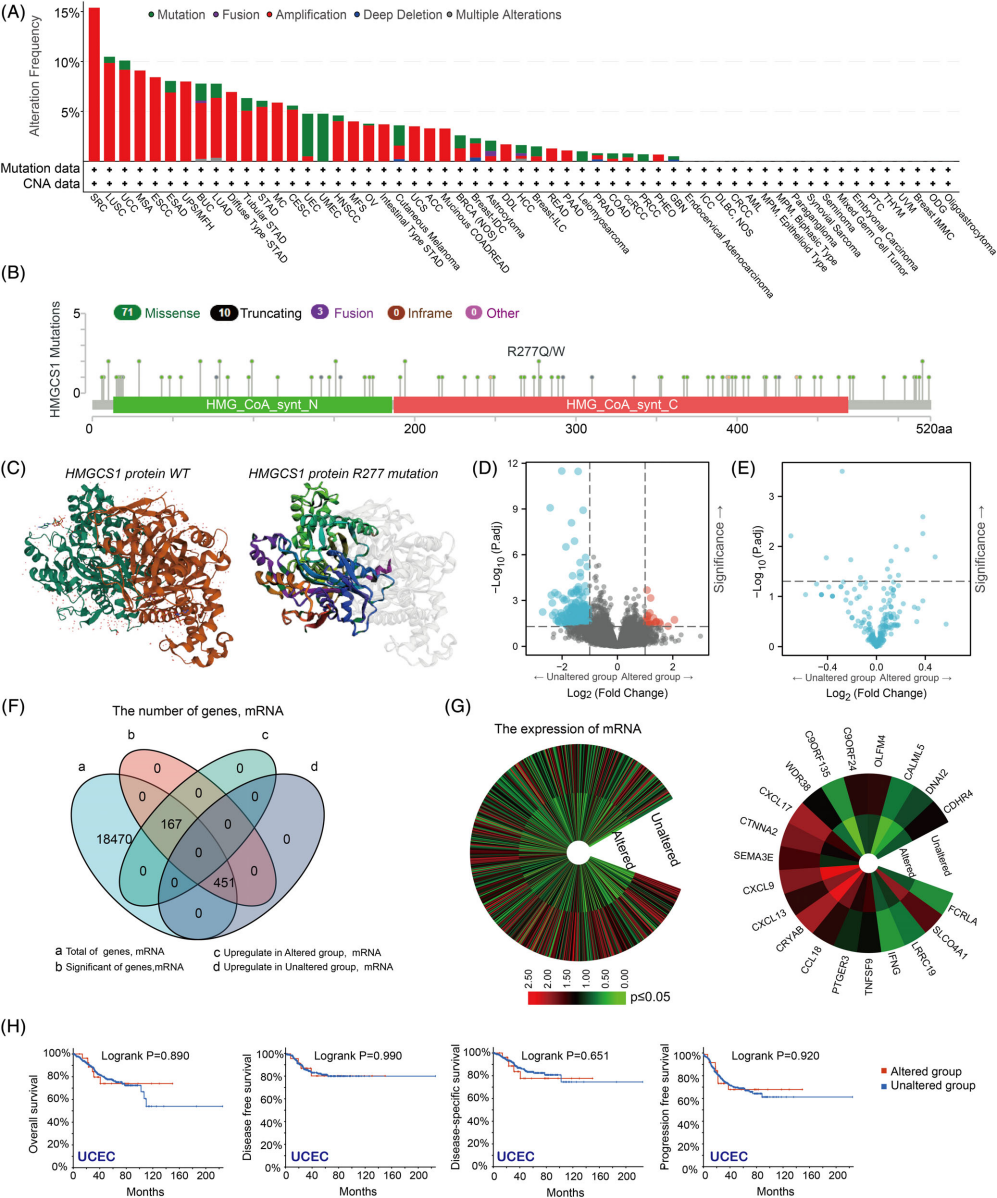
Genetic alterations of HMGCS1 in cancers. (A) The frequencies of various HMGCS1 genetic alterations in cancers (from cBioportal database). (B) Distribution of mutations on HMGCS1 protein sequence. (C) The 3D structures of HMGCS1 protein. Left panel: wildtype; right panel: R277 mutation. (D, E) The mRNA (D) and protein (E) expression levels of HMGCS1 in gene unaltered and altered groups of different cancers. (F) Number of genes with altered mRNA level in HMGCS1 unaltered and altered groups. (G) Gene expression levels in HMGCS1 unaltered and altered group (left panel). The top 20 differentially expressed genes between unaltered and altered group (right panel). (H) Overall, disease free, disease‐specific and progress free survival in cancers with or without HMGCS1 alterations, presented by Kaplan–Meier plots

We analyzed the genetic alterations of HMGCS1 in various tumors based on the TCGA dataset. We found that gene amplification (copy gain) was far more common than other forms of genetic alterations in HMGCS1, such as mutation, deep deletion (copy loss) and fusion (Figure 4A). Sarcoma had the highest HMGCS1 genetic alteration frequency, among which amplification was the primary type. It was noteworthy that the proportions of mutation were significant in UMEC (100%) and UEC (Figure 4A). Figure 4B displayed the types, distribution, and case numbers of HMGCS1 genetic alterations. Missense mutation was the most common type. The R277Q site mutation in the C‐terminal was detected in one case of UCEC, while R277W mutation was detected in one case of COAD. The R277Q/W site mutation resulted in a truncated form of HMGCS1 protein, impairing its function. Figure 4C showed the 3D structures of HMGCS1 with or without R277 mutation.

In addition, we aimed to study the relationship between HMGCS1 mutations and expression levels. We found that the mRNA levels were different between gene‐altered and gene‐unaltered group (Figure 4D). However, there is no significant difference of protein level between HMGCS1 altered and unaltered group (Figure 4E). The mRNA expression of total 618 genes, consisting of 167 up‐regulated genes in the gene‐altered group and 451 up‐regulated genes in the gene‐unaltered group, were associated with HMGCS1 mutation (Figure 4F). Left panel of Figure 4G displayed the difference of mRNA expression between altered and unaltered group. The top 20 differentially expressed genes were also shown (Figure 4G, right panel). Furthermore, we compared the OS, DFS, disease‐specific survival, and PFS between patients with and without genetic alterations. Kaplan–Meier analysis revealed that there was no significant difference UCEC (Figure 4H). Together, these data suggested that HMGCS1 mutation was correlated with alterations of transcriptome and proteome in tumors. However, R277 mutation had no significant effect on the prognosis of UCEC.

### 3.5 Enrichment analysis of HMGCS1‐related partners

**FIGURE 5:**
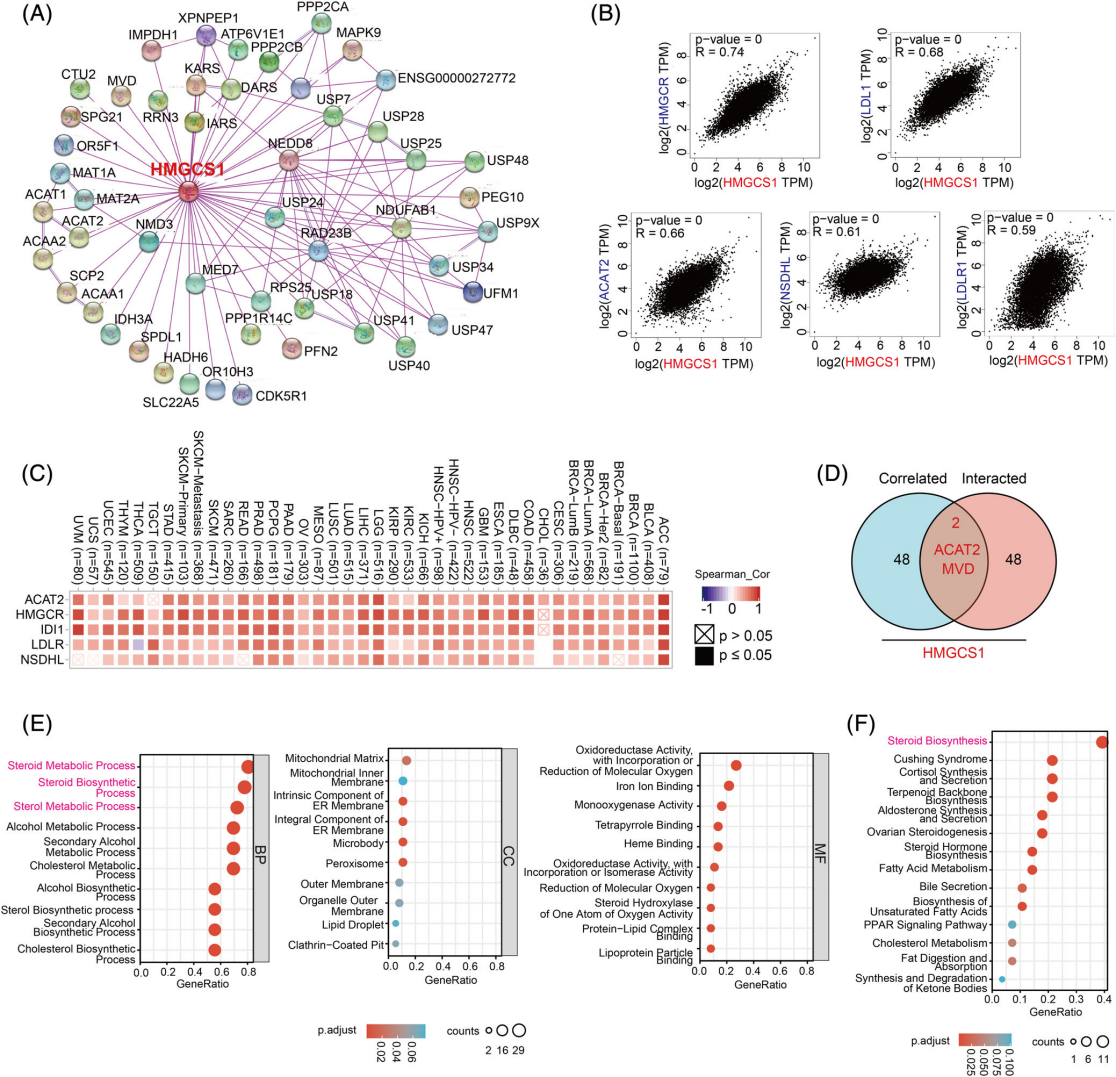
Enrichment analysis of HMGCS1‐related partners. (A) Protein–protein interaction network analysis based on STRING database. (B) The expression correlations between HMGCS1 and HMGCR, LDL1, ACAT2, NSDHL, and LDLR1, respectively. (C) The correlations in Figure 5B are further displayed with more details in different cancer types. (D) The intersection of HMGCS1‐binding and correlated genes. (E‐F) Functional enrichment analyses of HMGCS1 by GO (E) and KEGG (F). The top 10 terms in each analysis were listed. Color of dot represent adjusted p‐value. Size of dot represent number of genes of each term

**FIGURE 6:**
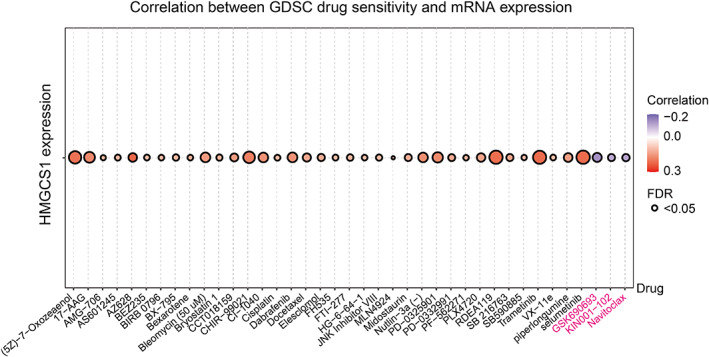
Analysis of the correlation between drug sensitivity and HMGCS1 expression using GDSC database. Figure summarized the top 30 drugs whose effects showed most significant associations with HMGCS1 expression in pan‐cancer. Only three drugs (GSK690693, KIN001‐102, and navitoclax) presented with negative correlation

Next, we tried to find the HMGCS1‐binding proteins as well as the HMGCS1 expression‐correlated genes, and further conducted pathway enrichment analyses. First, we searched the PPI networks using STRING online tools, and screened out fifty HMGCS1‐binding proteins with the closest relationship (Figure 5A). In addition, GEPIA2 was utilized to obtain the top 50 HMGCS1 expression correlated genes. The top five genes were HMGCR, LDL1 (low‐density lipoprotein 1), ACAT2 (acetyl‐CoA acetyltransferase 2), NSDHL (NAD[P] dependent steroid dehydrogenase‐like), and LDLR1 (low‐density lipoprotein receptor 1) (Figure 5B), all of which were positively correlated with HMGCS1 in most cancer types (Figure 5C). An intersection analysis of top 50 members from STRING and GEPIA2 showed two common members, namely ACAT2 and MVD (Figure 5D). Further, we utilized these 98 members to conduct KEGG and GO enrichment analyses to establish the functions of HMGCS1. GO analysis showed that the top three enriched terms in the biological process ontology were steroid metabolic process, steroid biosynthetic process, and sterol metabolic process. For the cellular component ontology, the top three enriched terms included mitochondrial matrix, mitochondrial inner membrane, and intrinsic component of endoplasmic reticulum membrane. For the molecular function ontology, the top three terms were oxidoreductase activity, with incorporation or reduction of molecular oxygen; iron ion binding; and monooxygenase activity (Figure 6E). KEGG pathway analysis disclosed involvement of HMGCS1 in steroid biosynthesis, Cushing syndrome, cortisol synthesis and secretion, and so forth (Figure 6F).

### 3.6 The prediction of the correlation between HMGCS1 expression and drug sensitivity

For drug sensitivity analysis, we conducted drug response predictions using the GDSC database. We assessed the correlation between HMGCS1 expression and IC50 values of molecules, and found that high HMGCS1 expression could reduce the drug sensitivity of most drugs. Interestingly, high expression of HMGCS1 enhanced the sensitivity to three drugs or small molecules, which included GSK690693, KIN001‐102, and navitoclax (Figure 6). These results indicated that HMGCS1 was a potential therapeutic target in cancer.

## 4 DISCUSSION AND CONCLUSION

Mounting evidence demonstrated a close link between HMGCS1 and the initiation and progression of various tumors.^14^, ^15^, ^16^, ^17^ To elucidate the general role of HMGCS1 in different tumors, we conducted a pan‐cancer analysis of HMGCS1 in 33 different cancers based on the data from the most comprehensive database available, such as TCGA and CPTAC databases. We analyzed the gene expressions and genetic alterations of HMGCS1 in various caner types, and explored the impact of HMGCS1 on disease survival, IF and drug sensitivity.

HMGCS1 was highly expressed in multiple tumor types, such as tumors originated from alimentary tract, breast, and uterus (Figure 1D). Correlations of increased HMGCS1 expression with a shortened survival were seen in most types of cancer (Figure 2). In BRCA, both HMGCS1 mRNA and protein level were significantly elevated (Figure 1D, F). High HMGCS1 expression was closely correlated with poor OS as well as PFS (Figure 2). In addition, there was a positive correlation between the HMGCS1 expression and the EIV of CAF (Figure 3). Consistently, Walsh et al identified HMGCS1 as a specific marker of cancer stem cell, whose expression strikingly indicated aggressive feature of breast cancer.^6^ Like BRCA, HMGCS1 expression was elevated and negatively associated with OS and PFS in tumors of uterus, including CESC and UCEC (Figure 1D and 2). In CESC, there was a significantly negative correlation between the HMGCS1 expression and IF of CD8+ T‐cells (Figure S3). Further study is warranted to understand more about the function of HMGCS1 in CESC and UCEC. Taken together, these data highlighted the oncogenic role of HMGCS1 in cancer.

Our data showed that HMGCS1 had increased mRNA expression in most alimentary tract related cancers, including COAD, ESCA, and READ, with a few exceptions like STAD (Figure 1D, E). For colon cancer, the HMGCS1 protein level was also elevated (Figure 1F). It is well known that RAS and RAF mutations are the most common genetic alteration in colon cancer (approximately 39%–53%), which are closely related to poor PFS, OS and nonresponse.^19^ Previously, we reported that HMGCS1 was a synthetical partner of BRAF^V600E^‐expressing colon and melanoma cancer cells.^14^ Sheng et al recently reported that HMGCS1 was overexpressed in colon cancer cells and tissues, promoting proliferation, migration, and invasion. Simultaneous targeting HMGCS1 could enhance the sensitivity of MEK inhibitor.^20^ Thus, targeting HMGCS1 could overcome RAF/RAS mutation mediated drug resistance, which might be a promising therapeutic strategy for patients with colon cancer. Notably, I‐Han et al. reported higher HMGCS1 expression in STAD tumor cells (*n* = 261) and metastatic lymph nodes (*n* = 26) compared to adjacent corresponding control tissues (*n* = 261) using q‐PCR method.^15^ However, in our study, the expression difference between control tissues (*n* = 32) and tumor tissues (*n* = 375) was not significant in STAD (*p* = 0.345), according to the TCGA data analysis (Figure 1D). The reason for the inconsistency may be the relatively small size of control sample in TCGA dataset. Interestingly, I‐Han et al. also discovered the nonmetabolic function of HMGCS1. Cytosolic HMGCS1 could translocate to nucleus and further bind and activated the promoters of downstream targets, contributing to the progression of gastric cancer.^15^ In conclusion, HMGCS1 is a promising target in alimentary tract related cancers.

It is also noteworthy that HMGCS1 may exert oncogenic function in tumors without overexpression. For instance, HMGCS1 was not overexpressed in prostate cancer cells but was in stroma. Through an autocrine/paracrine mechanism, the stromal cells with increased HMGCS1 expression could promote the growth of prostate cancer cells.^21^ Another example is AML. We have found that HMGCS1 was not significantly overexpressed in newly diagnosed AML patients. However, HMGCS1 expression was raised in refractory and relapsed AML, which played a vital role in reducing chemotherapy sensitivity (data not shown). More studies are required to understand the role of HMGCS1 in some tumors without elevated expression.

HMGCS1 was negatively correlated with both OS and DFS in LGG and KIRC. Currently, there is no report concerning about how HMGCS1 affects LGG. In KIRC, differential expression of HMGCS1 was reported by Huang et al..^22^ By analyzing data from TCGA, they identified HMGCS1 as one of good prognosis biomarkers.^22^ However, further research is needed to elucidate its role in KIRC.

Immune cells niched in the tumor microenvironment are closely linked to tumorigenesis.^23^ CAF, differentiated from stroma cells, are potent tumor‐promoting cells. Their proliferation was positively correlated with HMGCS1 expression in multiple cancer types. However, the proliferations of tumor‐antagonizing cells, such as CD8+ T‐cells, were overall negatively correlated with HMGCS1 level (Figure 3 and S2). These results indicated the role of HMGCS1 in modulating tumor‐infiltrating immune cells, which has not been reported yet and require further exploration.

As shown in Figure 4, amplification was the most frequent genetic alteration in tumors, which was consistent with previous data, which showed that HMGCS1 was overexpressed and closely related with prognosis in multiple tumor types including COAD and UCEC. Mutations were seen in several cancer types, though at a quite low frequency. We found R277Q mutation in one case of UCEC, and R277W mutation in one case of COAD. By further analysis in UCEC, we found that this missense mutation did not show prominent impact on OS, DFS, and PFS. By consulting the uniport website and literature,^24^, ^25^ we identified the active sites of HMGCS1, which included Glu95, Cys129, and His264, and the binding sites for Coenzyme A, which included Asn167, Ser221, Lys269, and Lys273. Currently, no mutation on these sites was found in tumors. The impact of R277Q mutation on prognosis was minimal, as the mutation seemed not to decrease the activity of HMGCS1. However, HMGCS1 mutation could cause obvious alterations in the expressions of multiple genes. It will be interesting to study the impact of these expression alterations on UCEC cancer cells.

As we can see in Figure 5, HMGCS1 was closely correlated and interacted with ACAT2 and MVD. All of three are involved in the MVA pathway and regulated by sterol regulatory element binding protein 2 (SREBP2).^26^ It has been reported that, after statin treatment, cancer cells could sustain the MVA metabolism by upregulating the expression of other genes in the MVA pathway via the feedback regulation of SREBP2.^27^ As MVA pathway is complicatedly intertwined, combined inhibition of multiple targets may enhance the cytotoxicity effects. Furthermore, enrichment analysis indicated a prominent role of HMGCS1 in steroid metabolism (Figure 5F). It is widely known that MVA‐derived steroids are the raw material for the synthesis of many gonadal steroid hormones, contributing to the development and progression of hormone dependent tumors such as prostate and breast cancer.^28^, ^29^ As mentioned before, several reports have demonstrated the oncogenic role of HMGCS1 in prostate and breast cancer. Besides, steroid biosynthesis has been shown to play a central role in T cells to achieve immune evasion.^30^ In conclusion, HMGCS1 is a promising target in cancer treatment.

High HMGCS1 expression could reduce the sensitivity of many drugs, indicating its potential role in drug resistance. However, HMGCS1 enhanced the sensitivity of GSK690693, KIN001‐102, and navitoclax (Figure 6). GSK690693 and KIN001‐102 are Akt inhibitors, while navitoclax is a BCL‐2 inhibitor. This finding sheds lights on the potential clinical application of these drugs to overcome HMGCS1 mediated drug resistance. Hymeglusin is the specific HMGCS1 inhibitor,^31^ which functions through binding to Cys‐129.^25^ We have proved its functions of suppressing cell growth and promoting apoptosis (data not shown). Further studies are required to know more about its functions and sides effects.

There were several limitations about our study. The evaluation of HMGCS1 alone may not recapitulate the whole characteristics of the MVA pathway. Therefore, it is important to further explore other vital genes and metabolites in this pathway to comprehensively uncover the function of MVA pathway in pan‐cancer. Additionally, a negative correlation between HMGCS1 expression and survival outcomes was presented after data analysis in this study. To make the conclusion more convincing, more basic and clinical research were needed. Furthermore, according to our and others previous reports, HMGCS1 also had impacts on cell proliferation, apoptosis, and immunity. The detailed mechanisms needed to be studied further.

Taken together, our pan‐cancer analysis provides a broad perspective of the oncogenic roles of HMGCS1 across different tumors and points out new directions for the treatment of cancer.

## CONFLICT OF INTEREST

The authors declare that they have no conflict of interest.

## AUTHOR CONTRIBUTIONS

C.Z.; Conceptualization (lead); data curation (lead); writing – original draft (equal). Z.W.; Conceptualization (equal); data curation (equal); funding acquisition (equal); writing – original draft (equal). Y.C.; Data curation (supporting). L.Z.; Conceptualization (supporting); funding acquisition (lead); supervision (lead); writing – review and editing (lead).

### ETHICS STATEMENT

This study is exempt from institutional review board approval.

## Supporting information


**Figure S1** Expression levels of HMGCS1 in different tumors and pathological stages. (A‐B) The HMGCS1 expression in AML, SKCM, LGG, OV, TGCT, and UCS. *P < 0.05. T: tumor tissues; N: normal tissues; AML: acute myeloid leukemia; SKCM: skin cutaneous melanoma; LGG: liver hepatocellular carcinoma; OV: ovarian serous cystadenocarcinoma; TGCT: testicular germ cell tumors; UCS: uterine carcinosarcoma. (C) The HMGCS1 mRNA levels in different cancer stages of KIRC, OV and THCA, which were analyzed by the Pathological Stage Plot module of GEPIA2. Log2 (TPM + 1) was applied for log‐scale. KIRC: kidney renal clear cell carcinoma; THCA: thyroid carcinoma.
**Figure S2**. The correlation between HMGCS1 expression level and OS (A), and DFS (B) in different tumors. OS: overall survival; DFS: disease‐free survival.
**Figure S3**. An illustration of immune component outputs upon a query to the immune components. (A) Correlation analysis between HMGCS1 expression and immune infiltration of CD8+ T‐cells across all types of cancer in TCGA. (B) Some representative scatter plots showed the potential correlation between HMGCS1 expression level and the infiltration level of CD8+ T‐cells.

## Data Availability

The data that support the findings of this study are available from the corresponding author upon reasonable request.
