# Supplementary material for: Pan‐cancer analysis reveals the oncogenic role of 3‐hydroxy‐3‐methylglutaryl‐CoA synthase 1
Source: Cancer Rep (Hoboken). 2021 Sep 22;5(9):e1562. doi: 10.1002/cnr2.1562 (PMC9458500; doi:10.1002/cnr2.1562)

**Figure S1. Expression levels of HMGCS1 in different tumors and pathological stages**. (A-B) The HMGCS1 expression in AML, SKCM, LGG, OV, TGCT, and UCS. *P<0.05. T: tumor tissues; N: normal tissues; AML: acute myeloid leukemia; SKCM: skin cutaneous melanoma; LGG: liver hepatocellular carcinoma; OV: ovarian serous cystadenocarcinoma; TGCT: testicular germ cell tumors; UCS: uterine carcinosarcoma. (C) The HMGCS1 mRNA levels in different cancer stages of KIRC, OV and THCA, which were analyzed by the Pathological Stage Plot module of GEPIA2. Log2 (TPM+1) was applied for log-scale. KIRC: kidney renal clear cell carcinoma; THCA: thyroid carcinoma.

**Figure S2.** The correlation between HMGCS1 expression level and OS (A), and DFS (B) in different tumors. OS: overall survival; DFS: disease-free survival.

**Figure S3.** **An illustration of immune component outputs upon a query to the immune components.** (A) Correlation analysis between HMGCS1 expression and immune infiltration of CD8+ T-cells across all types of cancer in TCGA. (B) Some representative scatter plots showed the potential correlation between HMGCS1 expression level and the infiltration level of CD8+ T-cells.


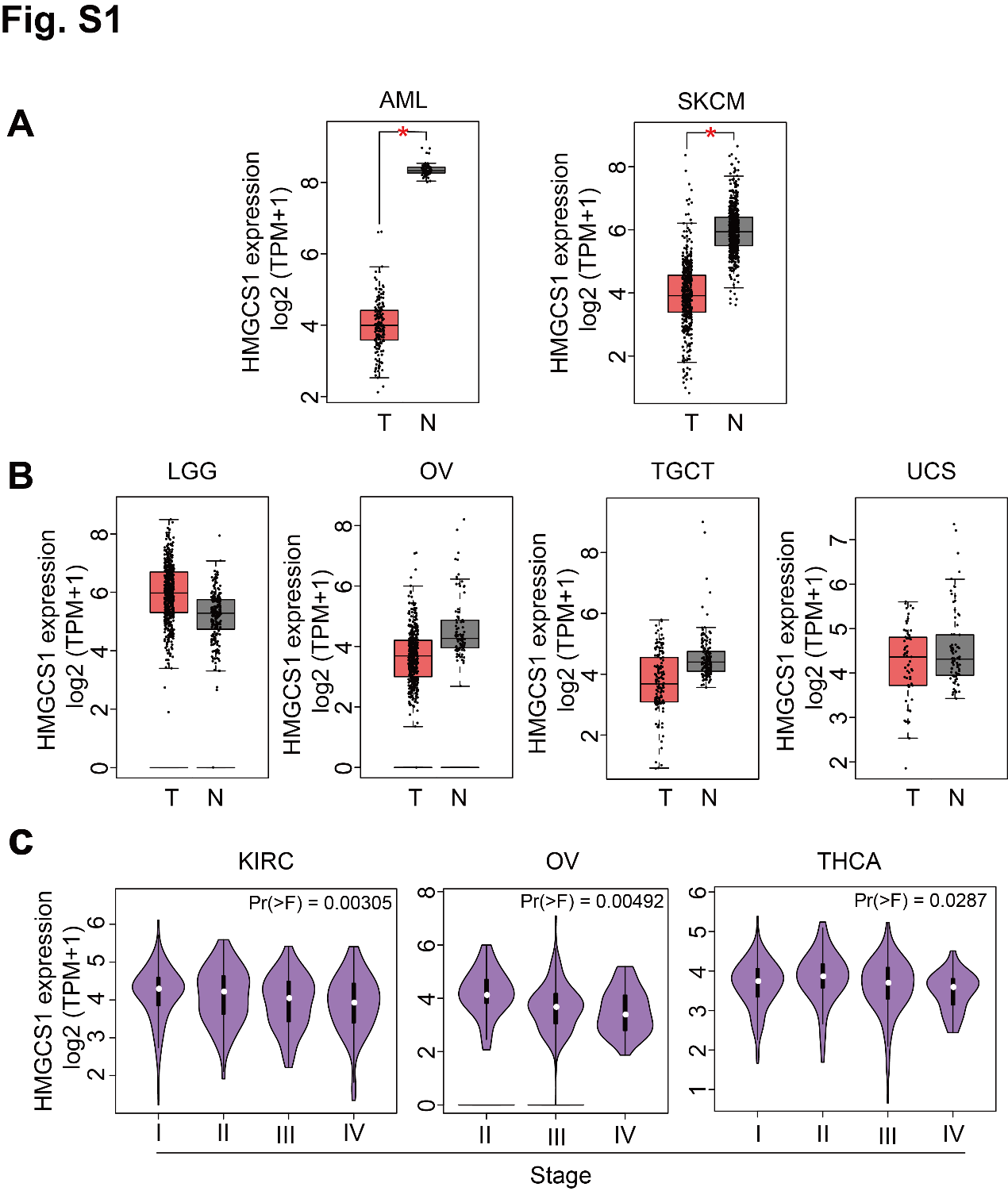


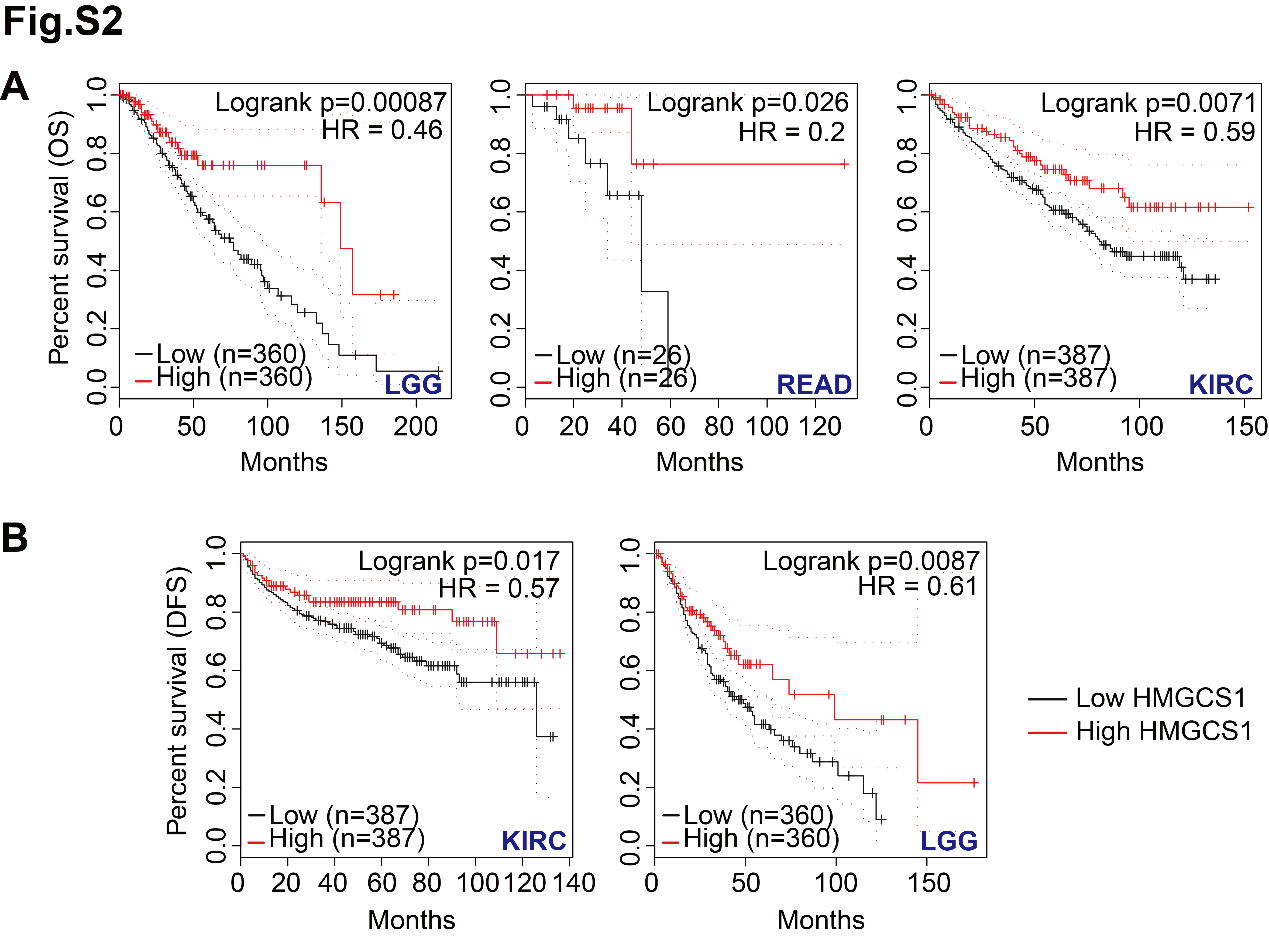


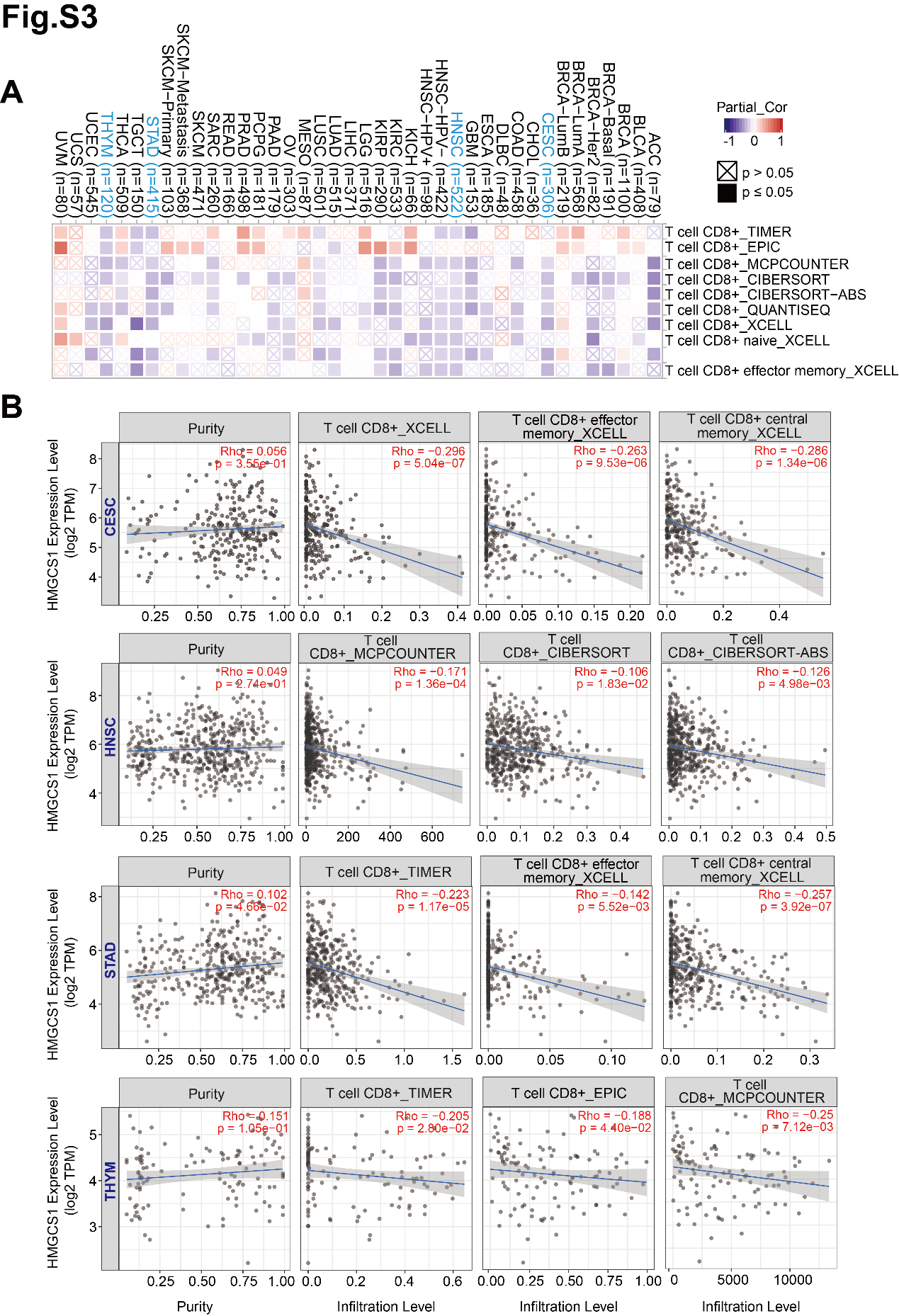

Supplement: Supplementary file 1 — Figure S1 Expression levels of HMGCS1 in different tumors and pathological stages. (A‐B) The HMGCS1 expression in AML, SKCM, LGG, OV, TGCT, and UCS. *P < 0.05. T: tumor tissues; N: normal tissues; AML: acute myeloid leukemia; SKCM: skin cutaneous melanoma; LGG: liver hepatocellular carcinoma; OV: ovarian serous cystadenocarcinoma; TGCT: testicular germ cell tumors; UCS: uterine carcinosarcoma. (C) The HMGCS1 mRNA levels in different cancer stages of KIRC, OV and THCA, which were analyzed by the Pathological Stage Plot module of GEPIA2. Log2 (TPM + 1) was applied for log‐scale. KIRC: kidney renal clear cell carcinoma; THCA: thyroid carcinoma. Figure S2. The correlation between HMGCS1 expression level and OS (A), and DFS (B) in different tumors. OS: overall survival; DFS: disease‐free survival. Figure S3. An illustration of immune component outputs upon a query to the immune components. (A) Correlation analysis between HMGCS1 expression and immune infiltration of CD8+ T‐cells across all types of cancer in TCGA. (B) Some representative scatter plots showed the potential correlation between HMGCS1 expression level and the infiltration level of CD8+ T‐cells. [file CNR2-5-e1562-s001.docx]
